# The Pacific Biosciences *de novo* assembled genome dataset from a parthenogenetic New Zealand wild population of the longhorned tick, *Haemaphysalis longicornis* Neumann, 1901

**DOI:** 10.1016/j.dib.2019.104602

**Published:** 2019-10-04

**Authors:** Felix D. Guerrero, Kylie G. Bendele, Noushin Ghaffari, Joseph Guhlin, Kristene R. Gedye, Kevin E. Lawrence, Peter K. Dearden, Thomas W.R. Harrop, Allen C.G. Heath, Yanni Lun, Richard P. Metz, Pete Teel, Adalberto Perez de Leon, Patrick J. Biggs, William E. Pomroy, Charles D. Johnson, Philip D. Blood, Stanley E. Bellgard, Daniel M. Tompkins

**Affiliations:** a USDA-ARS Knipling-Bushland US Livestock Insects Research Laboratory, Kerrville, TX, USA; b Texas A&M AgriLife, College Station, TX, USA; c Genomics Aotearoa and Biochemistry Department, University of Otago, Dunedin, New Zealand; d School of Veterinary Science, Massey University, Palmerston North, New Zealand; e AgResearch Ltd., c/o Hopkirk Research Institute, Private Bag 11008, Palmerston North, 4442, New Zealand; f Department of Entomology, Texas A&M University, College Station, TX, USA; g School of Fundamental Sciences, Massey University, Palmerston North, New Zealand; h Pittsburgh Supercomputing Center, Carnegie Mellon University, Pittsburgh, PA, USA; i Manaaki Whenua-Landcare Research, Auckland, New Zealand; j Department of Zoology, University of Otago, Dunedin, New Zealand

**Keywords:** Tick genome, Pac Bio *de novo* assembly, Genome annotation, Cattle tick

## Abstract

The longhorned tick, *Haemaphysalis longicornis*, feeds upon a wide range of bird and mammalian hosts. Mammalian hosts include cattle, deer, sheep, goats, humans, and horses. This tick is known to transmit a number of pathogens causing tick-borne diseases, and was the vector of a recent serious outbreak of oriental theileriosis in New Zealand. A New Zealand-USA consortium was established to sequence, assemble, and annotate the genome of this tick, using ticks obtained from New Zealand's North Island. In New Zealand, the tick is considered exclusively parthenogenetic and this trait was deemed useful for genome assembly. Very high molecular weight genomic DNA was sequenced on the Illumina HiSeq4000 and the long-read Pac Bio Sequel platforms. Twenty-eight SMRT cells produced a total of 21.3 million reads which were assembled with Canu on a reserved supercomputer node with access to 12 TB of RAM, running continuously for over 24 days. The final assembly dataset consisted of 34,211 contigs with an average contig length of 215,205 bp. The quality of the annotated genome was assessed by BUSCO analysis, an approach that provides quantitative measures for the quality of an assembled genome. Over 95% of the BUSCO gene set was found in the assembled genome. Only 48 of the 1066 BUSCO genes were missing and only 9 were present in a fragmented condition. The raw sequencing reads and the assembled contigs/scaffolds are archived at the National Center for Biotechnology Information.

**Table:** Specifications Table

**Subject**	Biology
**Specific subject area**	Genomics
**Type of data**	Assembled genome sequences and tables displaying sequencing, assembly, and repeats analysis statistics
**How data were acquired**	Long-read sequencing of very high molecular weight genomic DNA using Pacific Biosciences Sequel and Illumina HiSeq4000
**Data format**	Pacific Biosciences raw data in bam format, Illumina HiSeq4000 raw data in fastq format CANU-assembled Pacific Biosciences-only contigs/scaffolds in fasta format
**Parameters for data collection**	The expected large genome size of this tick necessitated the usage of long read sequencing technology and a genomic DNA isolation technique capable of purifying very high molecular weight DNA. The parthenogenetic nature of New Zealand populations of *Haemaphysalis longicornis* would provide more uniform genomic DNA, thus assisting the assembly of reads into contigs and scaffolds.
**Description of data collection**	Eggs from New Zealand-collected *H. longicornis* females were used to purify very high molecular weight genomic DNA, using a proteinase K/RNAse A/phenol-based extraction protocol. This DNA was sequenced on the Pacific Biosciences Sequel and Illumina HiSeq4000 platforms. The Sequel reads were assembled using CANU.
**Data source location**	Institution: Massey University, School of Veterinary Sciences, Palmerston North, New Zealand City/Town/Region: Gisborne, Whangara Country: New Zealand Latitude and longitude (and GPS coordinates) for collected samples/data:] Latitude: −38.545046 Longitude: 178.132273 GPS: −38.545046, 178.132273
**Data accessibility**	Repository name: National Center for Biotechnology Information Direct URL to data: https://www.ncbi.nlm.nih.gov/Data identification number: Raw read data is available at the National Center for Biotechnology Information (NCBI) Short Read Archive (SRA) through the SRA accession number SRR9226158 (Pacific Biosciences Sequel) and SRR9226159 (HiSeq4000). The whole genome shotgun assembly project has been deposited under the accession VFIB00000000. The version described in this paper is the first version, VFIB01000000. The overall BioProject ID is PRJNA540490 and the BioSample accession is SAMN11539514.

**Table:** 

**Value of the Data** • This assembled genome is the highest quality tick genome publicly available. • Researchers studying arachnid and tick genomics, arachnid evolution, and comparative genomics will find the assembled genome valuable. • The dataset can be used to study parthenogenesis-related genes, as this tick exclusively utilizes parthenogenetic reproduction in New Zealand. • The developers of novel tick control technologies for this and other species of ticks will find this genome very useful.

## 1 Data

*Haemaphysalis longicornis* is a three-host tick, with a wide distribution in temperate regions of Asia, Australia, and New Zealand [1]. This tick is capable of parthenogenetic reproduction, which allows rapid invasion of new areas and explosive population growth in established ranges. *Haemaphysalis longicornis* has recently established stable populations in several regions of the United States, and the tick's capacity for harboring and spreading several pathogens has heightened researchers interest in this tick [2]. Very high molecular weight genomic DNA was purified from eggs collected from parthenogenetic female *H. longicornis* ticks sourced from New Zealand. The genomic DNA was sequenced using 28 SMRT cells on Pacific Biosciences Sequel and 3 full lanes on the Illumina HiSeq4000 platforms. An all-Pac Bio reads genome was assembled using Canu. Raw read data is available at the National Center for Biotechnology Information (NCBI) Short Read Archive (SRA) through the SRA accession number SRR9226158 (Pacific Biosciences Sequel) and SRR9226159 (HiSeq4000). The whole genome shotgun assembly project has been deposited under the accession VFIB00000000. The version described in this paper is the first version, VFIB01000000. The overall BioProject ID is PRJNA540490 and the BioSample accession is SAMN11539514. The dataset contains the raw sequencing data and assembled genome of the tick. The data files were deposited at NCBI under project accession No. PRJNA540490. Information about the sequence reads, assembled genome, and genome repeats analysis is presented in Table 1, Table 2, Table 3, respectively.

**Table 1:** Statistics of the *H. longicornis* sequence reads.

Total SMRT cells	28
Total Subreads^a^	21,309,718
Overall Subread Mean length	11,671 bp
Total bp	248,705,718,800
Genome coverage^b^	83 X
Subread N50	9141 bp
Maximum SMRT cell Mean Subread length	13,705 bp
Minimum SMRT cell Mean Subread length	8739 bp

a These are reads ultimately used in the genome assembly.

b Based on estimated genome size of 3.0 Gb.

**Table 2:** General features of the *H. longicornis* genome assembly.

Contig/scaffold count	34,211
Mean contig/scaffold length	215,205 bp
Contig N50	515,769 bp (n = 3395)
Contig N90	85,735 (n = 17595)
Largest contig	8,678,875 bp
Total Length	7,362,387,268
GC Content	47.5%
Total BUSCO groups searched	1066
Number of BUSCO complete and single copy (% of total)	171 (16%)
Number of BUSCO complete and duplicated (% of total)	841 (79%)
Number of BUSCO fragmented (% of total)	8 (1%)
Number of BUSCO missing (% of total)	46 (4%)

**Table 3:** Repeat Modeller Analysis of the *H. longicornis* genome assembly.

Element ID	Number^a^	Total Length (bp)	Percent of genome^b^
SINEs	567,935	139,600,732	1.9
ALUs	0	0	0
MIRs	0	0	0
LINEs	1,123,711	811,749,916	11.03
LINE1	46,225	34,076,225	0.46
LINE2	75,136	40,371,988	0.55
L3/CR1	147,929	97,491,849	1.32
LTR elements	360,333	313,740,113	4.26
ERVL	0	0	0
ERVL-MaLRs	0	0	0
ERV class I	1469	67,439	0
ERV class II	4841	2,961,479	0.04
DNA elements	500,717	184,699,582	2.51
hAT-Charlie	22,723	6,351,868	0.09
TcMar-Tigger	64,096	21,409,145	0.29
Unclassified	6,206,150	1,961,583,750	26.64
Total interspersed repeats	8,758,846	3,411,374,093	46.34
Small RNA	505,644	114,045,117	1.55
Satellites	47,195	29,509,026	0.40
Simple repeats	1,629,716	133,804,303	1.82
Low complexity	86,281	4,409,140	0.06

a Most repeats fragmented by insertions or deletions have been counted as one element.

b Number of bases masked was 3,610,450,238 (49.04%).

## 2 Experimental design, materials, and methods

### 2.1 Tick tissue

Female ticks were collected by research staff from the Massey University School of Veterinary Sciences, removing them from cattle on a ranch at Whangara, near Gisborne, New Zealand during January 2018. Live females were maintained under ambient laboratory conditions and allowed to oviposit. Approximately 1 g of eggs was obtained, incubated under ambient laboratory conditions for 4 weeks, and then frozen at −80 °C.

### 2.2 Genomic DNA isolation and sequencing

A protocol from Sambrook et al. [3] was used to purify very high molecular weight genomic DNA from the eggs [4]. The protocol consisted of pulverizing frozen material in a liquid nitrogen-cooled mortar and pestle, addition to an aqueous buffer, followed by RNAse treatment, digestion by proteinase K, phenol extraction, and dialysis in 50 mM Tris, 10 mM EDTA, pH 8.0. The resultant DNA was determined by agarose gel electrophoresis to be > 200 kb. The DNA was concentrated for sequencing using Centricon Plus 70 Centrifugal Filter Units (Molecular Weight Cut Off = 3000; Millipore Sigma, Burlington, MA, USA) and 3 washes of approximately 50 ml wash buffer (50 mM Tris, 10 mM EDTA, pH 8.0), centrifuging at 2500×*g* and 8 °C. Ten ml of this buffer was used to recover a final total of 0.4 mg of purified genomic DNA at a concentration of 37 mg/ml.

### 2.3 Assembly and analysis

Sequencing was performed at the Texas A&M AgriLife Genomics and Bioinformatics Service, College Station, TX using 28 SMRT cells on the Pacific Biosciences Sequel and 3 lanes of the Illumina HiSeq4000 platforms. Read quality checks and filtering of raw reads were conducted via the manufacturer's standard protocol and protocols developed at the Texas A&M AgriLife Genomics and Bioinformatics Service prior to submission to NCBI and assembly. The original intent was to use the Illumina reads to error-correct the Sequel long reads. However, due to the high amount of required computational resources necessary to error-correct and assemble large genomes, we chose to create a Sequel-only assembly using the Canu [5] pipeline. We hypothesized the parthenogenetic nature of New Zealand's *H. longicornis* populations [1] would minimize genomic DNA heterogeneity and allow for a high-quality Pacific Biosciences-only genome assembly.

We utilized allocations on the Pittsburgh Supercomputing Center *Bridges* system [6], granted through the Extreme Science and Engineering Discovery Environment (XCEDE) program sponsored by the National Science Foundation [7]. The Canu assembly took over 24 consecutive days, running on a reserved node with access to 352 cores, 12 TB of RAM, and node-local disk storage to avoid unnecessary data transfers. Program parameters were corMhapSensitivity = high, corOutCoverage = 100, batOptions = -dg3 -db 3 -dr 1 -ca 500 -cp 50, and an input genome size assumption of 3 Gb, estimated from our experience with *Rhipicephalus* tick genomes. The Canu assembly output estimated genome size to be 7.36 Gb and we are working to verify this result using independent genome size determination protocols. When the 34,211 assembled genome contigs were submitted to NCBI for archiving, only four contigs were detected to have contaminating sequence and those contaminations were corrected by NCBI staff. BUSCO (v. 3.0.2) analysis was run on the assembled genome against the arthropoda BUSCO set, using AUGUSTUS fly pre-configured prediction model with arguments -m genome -sp fly -c 8 [8]. Statistics from the sequencing are in Table 1 while features of the assembled genome are in Table 2. *De novo* repeats were identified with RepeatModeler v. 1.0.11 [9] using the NCBI engine (BLAST + software v. 2.8.1) and then masked using Repeat Masker v. 4.0.9 [10] using a combined repeat database of classified repeats from RepeatModeler, the *ticks* library included as part of RepeatMasker, Dfam 3.0, and RepBase-20170127, using the following parameters: *-e ncbi -gccalc -frag 2000000 -qq -xsmall*. The results from the repeats analysis are shown in Table 3.
